# Diagnostic implications of a small-voxel reconstruction for loco-regional lymph node characterization in breast cancer patients using FDG-PET/CT

**DOI:** 10.1186/s13550-018-0359-7

**Published:** 2018-01-16

**Authors:** Daniëlle Koopman, Jorn A. van Dalen, Hester Arkies, Ad H. J. Oostdijk, Anne Brecht Francken, Jos Bart, Cornelis H. Slump, Siert Knollema, Pieter L. Jager

**Affiliations:** 1 Department of Nuclear Medicine, Isala, Zwolle, the Netherlands; 2 MIRA Institute for Biomedical Technology and Technical Medicine, University of Twente, Enschede, the Netherlands; 3 Department of Medical Physics, Isala, Zwolle, the Netherlands; 4 Department of Surgery, Isala, Zwolle, the Netherlands; 5 Department of Pathology, Isala, Zwolle, the Netherlands

**Keywords:** State-of-the-art PET/CT, Small-voxel reconstruction, Breast cancer, Loco-regional lymph nodes

## Abstract

**Background:**

We evaluated the diagnostic implications of a small-voxel reconstruction for lymph node characterization in breast cancer patients, using state-of-the-art FDG-PET/CT. We included 69 FDG-PET/CT scans from breast cancer patients. PET data were reconstructed using standard 4 × 4 × 4 mm^3^ and small 2 × 2 × 2 mm^3^ voxels. Two hundred thirty loco-regional lymph nodes were included, of which 209 nodes were visualised on PET/CT. All nodes were visually scored as benign or malignant, and SUV_max_ and TB_ratio_(=SUV_max_/SUV_background_) were measured. Final diagnosis was based on histological or imaging information. We determined the accuracy, sensitivity and specificity for both reconstruction methods and calculated optimal cut-off values to distinguish benign from malignant nodes.

**Results:**

Sixty-one benign and 169 malignant lymph nodes were included. Visual evaluation accuracy was 73% (sensitivity 67%, specificity 89%) on standard-voxel images and 77% (sensitivity 78%, specificity 74%) on small-voxel images (*p* = 0.13). Across malignant nodes visualised on PET/CT, the small-voxel score was more often correct compared with the standard-voxel score (89 vs. 76%, *p* <  0.001). In benign nodes, the standard-voxel score was more often correct (89 vs. 74%, *p* = 0.04).

Quantitative data were based on the 61 benign and 148 malignant lymph nodes visualised on PET/CT. SUVs and TB_ratio_ were on average 3.0 and 1.6 times higher in malignant nodes compared to those in benign nodes (*p* <  0.001), on standard- and small-voxel PET images respectively. Small-voxel PET showed average increases in SUV_max_ and TB_ratio_ of typically 40% over standard-voxel PET. The optimal SUV_max_ cut-off using standard-voxels was 1.8 (sensitivity 81%, specificity 95%, accuracy 85%) while for small-voxels, the optimal SUV_max_ cut-off was 2.6 (sensitivity 78%, specificity 98%, accuracy 84%). Differences in accuracy were non-significant.

**Conclusions:**

Small-voxel PET/CT improves the sensitivity of visual lymph node characterization and provides a higher detection rate of malignant lymph nodes. However, small-voxel PET/CT also introduced more false-positive results in benign nodes. Across all nodes, differences in accuracy were non-significant. Quantitatively, small-voxel images require higher cut-off values. Readers have to adapt their reference standards.

## Background

In recent years, there has been an increasing role for fluorine-18 fluordeoxyglucose positron emission tomography (FDG-PET) combined with computed tomography (CT) in the diagnostic evaluation of patients with stage II–IV primary breast cancer [1, 2]. FDG-PET/CT has now largely replaced conventional staging that included a bone scan, abdominal echography and chest x-ray. This is due to PET/CT’s higher accuracy and the ability to perform whole-body staging in a single session [1, 3, 4].

In patients with stage II–IV primary breast cancer, accurate detection of loco-regional lymph nodes and distant metastasis is crucial for treatment selection and prognosis prediction. However, the sensitivity and detection rate of small lesions and lesions with low metabolism using FDG-PET are restricted, due to the limited PET spatial resolution [5, 6]. For axillary lymph node staging using FDG-PET/CT, a systematic review based on seven studies found an average sensitivity of 56% with 93% specificity [7]. More specifically, for micro-metastatic lesions (diameter ≤ 2 mm), a sensitivity of 11% was reported, while for macro-metastatic lesions (diameter > 2 mm), the sensitivity was 57% [7].

A PET reconstruction setting that possibly improves small lesion detection and sensitivity is the voxel size. In current practice, the image voxel size for whole-body FDG-PET scans is typically around 4 × 4 × 4 mm^3^ [8]. However, it has been suggested that in combination with new highly sensitive time of flight (TOF) PET/CT cameras, the use of reconstructions with smaller voxels might further improve the detection of small lesions [8, 9]. In a previous study, we have assessed to what extent small lesion detectability is influenced by the voxel size [10]. With the use of small 2 × 2 × 2 mm^3^ voxels, we found a profound increase in the standardized uptake value (SUV) and an improvement in signal-to-noise ratio (SNR) for small lesions, as compared to the values on PET images reconstructed with default 4 × 4 × 4 mm^3^ voxels.

In clinical trials that assessed the value of FDG-PET/CT in primary breast cancer patients scheduled for neo-adjuvant chemotherapy, the use of small-voxels for loco-regional lymph node characterization has been reported already [11, 12]. However, those studies did not compare standard- and small-voxel reconstructions in a clinical setting. Therefore, the diagnostic implications and potential clinical benefit of small 2 × 2 × 2 mm^3^voxels, in terms of small lesion detection and sensitivity, remain unknown. The purpose of this study is to evaluate the diagnostic implications of a small-voxel reconstruction for loco-regional lymph node characterization in breast cancer patients, using state-of-the-art FDG-PET/CT.

## Methods

### Inclusion

In this study, we have included 69 consecutive women with primary stage II–IV ductal primary breast cancer who had been referred for a pre-treatment whole-body FDG-PET/CT scan. We only included patients with at least one loco-regional lymph node which could be classified as benign or malignant based on histological or imaging information. We received a waiver from the Medical Ethical Committee of our institution (METC Isala, Zwolle) to perform this retrospective study, as it deals with an evaluation of clinically indicated scans. Informed consent was obtained from all individual participants included in the study.

### PET/CT data acquisition

Patients fasted for at least 6 h prior to scanning. Before intravenous injection of FDG, blood glucose levels were measured to ensure a value below 10 mmol/L. The mean glucose level was 5.4 mmol/L (range 3.8–9.3 mmol/L). A dedicated dose protocol depending quadratically on patients’ body weight was used. This protocol is described by the formula *A* = 3.8 × *w*^2^/*t*, where *A* is the FDG activity to administer (in megabecquerel), *w* is the patients’ body weight (in kilogram), and *t* is the acquisition time per bed position (in seconds). This approach has been shown to result in an image quality that does not depend on patient’s weight [13]. Acquisition times for the patient studies were 1 and 2 min per bed position for patients with body weight ≤ 80 and > 80 kg, respectively. The average administered FDG activity was 331 MBq (range 155–533 MBq).

All scans were acquired with patients in supine position, using a state-of-the-art PET/CT scanner (Ingenuity TF, Philips Healthcare, Cleveland, OH, USA). This fully three-dimensional TOF PET scanner is combined with a 128-slice CT scanner. The PET scan was acquired 60 min post-injection, using a whole-body protocol. Before PET imaging, a CT scan was acquired for attenuation correction. The CT scan parameters were tube voltage 120 kV, dose modulation with an average tube current of 53 mA (range 37–94 mA), slice collimation 64 × 0.625 mm, pitch 0.83 and rotation time 0.5 s.

### PET/CT data reconstruction

PET data were reconstructed using a list-mode TOF algorithm and line-of-response row-action maximum-likelihood algorithm method [14, 15], called BLOB-OS-TF. Images were reconstructed in two types of matrices: 144 × 144 matrices with voxel size 4 × 4 × 4 mm^3^ (standard-voxels) and 288 × 288 matrices with voxel size 2 × 2 × 2 mm^3^ (small-voxels). For the standard-voxel reconstruction, the blob had a 2.5-mm radius with a blob shape parameter of 8.4 mm. The blob radius and shape parameter for the small-voxel reconstruction were 2.8 and 6.4 mm, respectively. Furthermore, the relaxation parameters for the standard- and small-voxel reconstructions were 1.0 and 0.5, respectively. For both types of voxel reconstructions, 3 iterations and 43 subsets were applied. All reconstruction parameters were default settings recommended by the vendor. Point-spread function modelling was not applied.

CT data were reconstructed using an iterative reconstruction algorithm (iDose, Philips Healthcare, Cleveland, OH, USA) with iDose level 4 and a slice thickness of 3 mm. The administered FDG activity and PET/CT acquisition protocols were consistent with European Association of Nuclear Medicine (EANM) guidelines for tumour PET imaging [16, 17]. Moreover, the reconstructed PET images with standard-voxels fulfilled the EANM research Ltd. (EARL) accreditation specifications [18]. The small-voxel reconstruction does not fulfil the EARL accreditation specifications, because the recovery curves for the small 10- to 13-mm spheres increase up to values above the maximum EARL specifications [10].

### Visual evaluation

Integrated PET/CT data were reviewed on a dedicated workstation (IntelliSpace Portal 6, Philips Healthcare, Cleveland, OH, USA). First, each PET/CT scan was evaluated by two nuclear medicine (NM) physicians, with more than 5 years of experience in PET/CT viewing. They were blinded to the patient record and histological information and interpreted the PET/CT data by simultaneous viewing of PET, CT and fused PET/CT images. Both the standard- and small-voxel images were evaluated blindly, both separately and randomly. The NM physicians scored all loco-regional lymph nodes showing focal FDG-uptake on the standard- or small-voxel images. They integrated their PET reading with the presence, absence, shape and size of lymph nodes on the low-dose CT scan, in an identical fashion as used in clinical interpretation.

Initially, each lymph node was scored using a five-point ordinal scale with 1: certainly benign, 2: probably benign, 3: equivocal, 4: probably malignant and 5: certainly malignant. If this initial interpretation between both physicians differed, consensus was reached. This was needed for 39 lymph nodes (19%) on standard-voxel images and for 43 lymph nodes (21%) on small-voxels images. Next, to be able to evaluate the lymph node characterization performance, each lesion was assigned as benign or malignant using the following method. All lymph nodes with a score of 1 or 2 were allocated as benign. All lymph nodes with scores of 4 and 5 were allocated as malignant. Lymph nodes with a score of 3 were once again evaluated on the PET/CT images, and they received an ultimate score as benign or malignant.

### Quantitative evaluation

All scored lymph nodes were evaluated semi-quantitatively by an experienced PET reader blinded to the patient record, histological information and visual PET/CT scores. The maximum standardized uptake value (SUV_max_) was derived on the axial slice that contained the highest FDG-uptake of the lesion.

Next, we calculated the lymph node-to-background ratio (TB_ratio_), defined as the ratio between the lymph node SUV_max_ and the average SUV in the background (SUV_background_). To measure the SUV_background_, we defined two regions of interest (ROI1 and ROI2) on the axial PET image. ROI1 enclosed both the lymph node under study and a surrounding background area of 800 mm^2^, while ROI2 only enclosed the lymph node. For both ROIs, the area size and the average SUV (SUV_mean_) were collected to calculate the SUV_background_ in a donut-shaped ROI using Formula 1:

1 $$ {\mathrm{SUV}}_{\mathrm{background}}=\frac{\left(\mathrm{ROI}1\ {\mathrm{SUV}}_{\mathrm{mean}}\bullet \mathrm{ROI}1\ \mathrm{area}\right)-\left(\mathrm{ROI}2\ {\mathrm{SUV}}_{\mathrm{mean}}\bullet \mathrm{ROI}2\ \mathrm{area}\right)\ }{\mathrm{ROI}1\ \mathrm{area}-\mathrm{ROI}2\ \mathrm{area}} $$

Finally, for all the scored lymph nodes, we measured the short-axis diameter on the axial slice of the attenuation CT scan.

### Final diagnosis

**Fig. 1:**
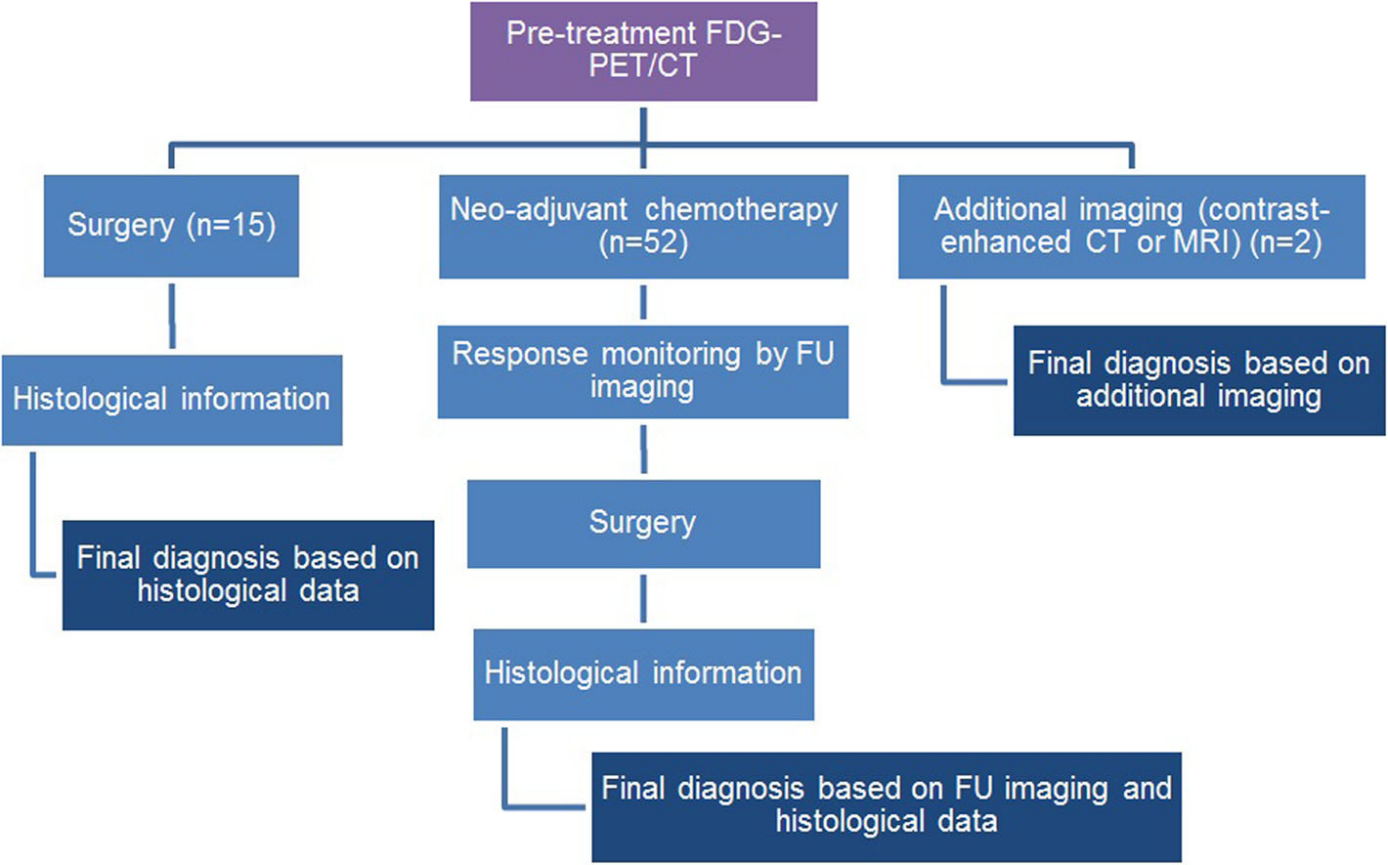
Flow chart showing the method to derive the final diagnosis per patient (*n*)

The final diagnosis for each lymph node was based on histological information, follow-up (FU) imaging (FDG-PET/CT, contrast-enhanced CT or magnetic resonance imaging (MRI)) or additional imaging (contrast-enhanced CT or MRI) in the following way (Fig. 1). For patients who initially underwent a surgical resection that included sentinel lymph node biopsy or axillary lymph node dissection, the final diagnosis was based on the histological information obtained during surgery. Pathology examination was part of the clinical evaluation and was centralised at our institution. Lymph nodes were histologically processed by formalin fixation followed by paraffin embedding, according to standardized procedures.

The lymph nodes were serially sectioned at 250 μm at three levels and stained with both hematoxylin and eosin, with an immune-histochemical cytokeratin staining (panCK). The immune-histochemical procedure was performed by a fully automated procedure, using pre-diluted antibodies on the Ventana Benchmark system (Roche Ventana, Tucson AZ, USA). The sizes of the metastases were measured on a conventional bright-field microscope (Leica, DM4000, Leica microsystems Germany) using a micro-measuring scale on glass slide (definition = 0.1 mm). In all lymph nodes, the largest diameter of a metastasis was reported.

For patients who were treated with neo-adjuvant chemotherapy, the final diagnosis was based on the response to therapy as visualised on FU imaging combined with the histological information that was available from the subsequent surgical resection. For these patients, lesions were considered malignant when they showed a decrease in size or FDG-uptake induced by subsequent chemotherapy. Furthermore, lesions that were stable in size and FDG-uptake during neo-adjuvant therapy were considered to be benign unless there was proof of malignancy from histological information obtained during surgery. When histological information or FU imaging was not available, the final diagnosis was based on the results of additional contrast-enhanced CT or MRI.

Additionally, we collected information on all malignant loco-regional lymph nodes that were found during surgical resection but which had not been visualised on FDG-PET or the attenuation CT. For those lesions, we recorded the metastatic deposit size that was measured during a separate pathology examination, performed by one pathologist (JB). For visual PET performance evaluation, these lymph nodes were regarded as benign nodes on PET. Furthermore, for quantitative PET evaluation, these lymph nodes were not taken into account because it was not possible to perform measurements on PET images.

### Lymph node characterization

Visual evaluation scores were analysed on a lesion-per-lesion basis, by comparing the scores on standard- and small-voxel images for each lymph node. Quantitatively, we calculated average values for SUV_max_ and TB_ratio_ in both benign and malignant lymph nodes and for both voxel reconstructions. We created receiver operator curves (ROC) and calculated the area under the curve (AUC) with a 95% confidence interval (CI) for SUV_max_ and TB_ratio_. For both reconstruction methods, we determined the sensitivity, specificity and accuracy for lymph node characterization from the visual and quantitative PET/CT evaluation, using the final diagnosis as a reference standard. We calculated optimal cut-off values for SUV_max_ and TB_ratio_ to distinguish benign from malignant lymph nodes on both voxel reconstructions_._ These cut-off values were based on the highest combined sensitivity and specificity (highest sum).

### Statistical analysis

We used the McNemar test for paired samples to compare the visual scores for both reconstructions with the final diagnosis. Quantitative results were presented as mean ± standard deviation (SD). We included ranges in uptake values and lymph node size. Differences in SUV_max_ and TB_ratio_ between benign and malignant lymph nodes were evaluated using the Mann-Whitney *U* test. Furthermore, to evaluate differences in characterization performances between standard and small-voxels for SUV_max_ and TB_ratio_, we compared the AUCs using a chi-square test. Additionally, the characterization performances for SUV_max_ and TB_ratio_ using optimal cut-off values were evaluated with the McNemar test for paired samples. A *p* value less than 0.05 was considered to indicate statistical significance.

## Results

### Patient characteristics

Clinical data from 69 patients are shown in Table 1. In total, 230 loco-regional lymph nodes were investigated (mean 3 ± 2 lymph nodes per patient). We have included 61 benign lymph nodes with an average size of 6 mm (range 3–12 mm), and 169 malignant lymph nodes with an average size of 8 mm (range 1–32 mm). During surgical resection in 11 patients, 21 malignant lymph nodes were found that were not visualised on PET/CT images. This group consisted of 6 micro-metastases (diameter ≤ 2 mm) and 15 macro-metastases with sizes varying between 2 and 7 mm diameter. The remaining 209 loco-regional lymph nodes, which were visualised on PET/CT, were visually and quantitatively evaluated in this study.

**Table 1:** General characteristics

Patient characteristics (*n* = 69)	Age (years)	53 ± 12 (mean ± SD)
	Body weight (kilogram)	76 ± 14
Hormonal receptor status	Oestrogen	51 pos., 16 neg., 2 unknown
	Progesterone	37 pos., 30 neg., 2 unknown
	Human epidermal growth factor receptor 2	19 pos., 48 neg., 2 unknown
Loco-regional lymph nodes (*n* = 230)	Location	
	Left region	108
	Right region	122
	Final diagnosis	
	Benign	61
	Malignant	169
	Final diagnosis based on the following:	
	Histological proof	128 (56%)
	FU imaging	99 (43%)
	Additional imaging	3 (1%)

*SD* standard deviation, *pos.* positive, *neg.* negative, *FU* follow-up

### Visual evaluation

For 173 out of 209 lymph nodes (83%), visual interpretation scores were exactly similar for both small and standard-voxels. Furthermore, 32 lymph nodes (15%) were scored malignant on the small-voxel images but benign on the standard-voxel images. Contrarily, four lymph nodes (2%) were scored benign on the small-voxel images but malignant on the standard-voxel images.

In Table 2, the final diagnosis of each lymph node is compared with the visual scores on both voxel reconstructions. Sensitivity, specificity and accuracy for standard-voxel PET/CT images in visual evaluation were 67, 89 and 73%, respectively. For small-voxel PET/CT images, we found a sensitivity, specificity and accuracy of 78, 74 and 77%, respectively. Across all lymph nodes, the differences in accuracy were not statistically significant (*p* = 0.13).

**Table 2:** Table comparing the final diagnosis with standard-voxel or small-voxel PET/CT visual scores for malignant lymph nodes, benign lymph nodes and all lymph nodes

	Small-voxel correct	Small-voxel not correct	*p* value
Malignant lymph nodes (*n* = 148)			
Standard-voxel correct	112	1	< 0.001
Standard-voxel not correct	20	15	
Benign lymph nodes (*n* = 61)			
Standard-voxel correct	42	12	0.04
Standard-voxel not correct	3	4	
All lymph nodes (*n* = 209)			
Standard-voxel correct	154	13	0.13
Standard-voxel not correct	23	19	

For malignant lymph nodes, the small-voxel score was more often correct as compared to the standard-voxel score (*p* < 0.001), while for benign lymph nodes, the standard-voxel score was more often correct (*p* = 0.04). Across all lymph nodes visualised on PET/CT, accuracies of standard- and small-voxel scores were comparable (*p* = 0.13)

*PET/CT* positron emission tomography/computed tomography

Limiting this analysis to the 148 malignant lymph nodes that were visualised on PET/CT, the small-voxel score was correct in 132 cases (89%) while the standard-voxel score was correct in 113 cases (76%), *p* < 0.001. In benign lymph nodes (*n* = 61) only, the small-voxel score was correct in 45 cases (74%) vs. 54 correct scores (89%) on standard-voxel images (*p* = 0.04).

### Quantitative evaluation

SUV_max_ and TB_ratio_ across all lymph nodes are shown in Table 3. For both SUV_max_ and TB_ratio_, and in both types of voxel reconstructions, uptake values for malignant lymph nodes were averagely a factor of 3.0 and 1.6 higher, respectively, as compared to those for benign nodes (*p* < 0.001). Furthermore, the use of small-voxels resulted in SUV increases of typically 40% (Table 3).

**Table 3:** SUV_max_ and TB_ratio_ for benign and malignant lymph nodes as measured on standard- and small-voxel PET images

		Benign lymph nodes (*n* = 61)	Malignant lymph nodes (*n* = 148)
SUV_max_	Standard-voxels	1.1 ± 0.4 (mean ± SD)	4.4 ± 3.3
	Small-voxels	1.5 ± 0.5	5.9 ± 4.1
	Percent change	37%	40%
TB_ratio_	Standard-voxels	2.0 ± 0.7	5.3 ± 4.2
	Small-voxels	2.8 ± 1.4	7.3 ± 5.2
	Percent change	44%	43%

SUV_max_ and TB_ratio_ for malignant lymph nodes were averagely 3.0 and 1.6 times as high as compared to benign nodes for both types of voxel reconstructions (*p* < 0.001). Mean SUV_max_ and TB_ratio_ typically increased with 40% when using small-voxels (*p* < 0.001)

*SUV*_*max*_ maximum standardized uptake value, *TB*_*ratio*_ ratio between the lymph node SUV_max_ and the lymph node background uptake, *PET* positron emission tomography, *SD* standard deviation

### Lymph node characterization by quantitative evaluation

**Fig. 2:**
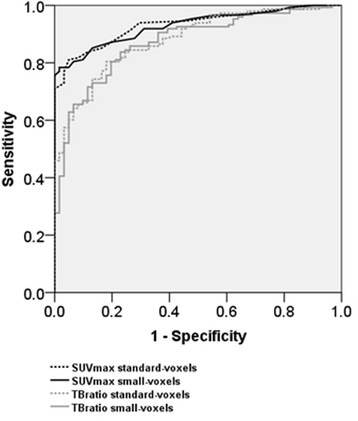
ROC curves for lymph node characterization using SUV_max_ and TB_ratio_, measured on standard- and small-voxel PET images. AUCs for SUV_max_ were 0.93 (95% CI 0.90–0.97) and 0.93 (95% CI 0.90–0.96) for standard- and small-voxels, respectively (*p* = 0.71). AUCs for TB_ratio_ were 0.88 (95% CI 0.84–0.93) and 0.87 (95% CI 0.82–0.92) for standard- and small-voxels respectively (*p* = 0.61). AUCs for SUV_max_ were significantly higher as compared to AUCs for TB_ratio_, for both standard- and small-voxels (*p* = 0.003 and *p* = 0.002)

Table 4 shows the sensitivity, specificity and accuracy of lymph node characterization for standard- and small-voxel PET images, using optimal cut-off values for SUV_max_ and TB_ratio_. Furthermore, receiver operator curves (ROC) for SUV_max_ and TB_ratio_ are shown in Fig. 2. TB_ratio_ had a significantly lower AUC as compared to the SUV_max_ parameter, for both standard and small-voxels (*p* = 0.003 and *p* = 0.002). AUC values for standard- and small-voxel images were comparable for both SUV parameters, with *p* = 0.71 for SUV_max_ and *p* = 0.61 for TB_ratio_. Additionally, no significant differences were found in characterization performances based on the accuracy between standard- and small-voxel images, with *p* = 0.11 for SUV_max_ and *p* = 0.29 for TB_ratio_. Table 4 shows that the use of small-voxels required higher SUV cut-offs for accurate lymph node characterization.

**Table 4:** Sensitivity, specificity and accuracy for SUV_max_ and TB_ratio_ at optimal cut-offs, determined for standard- and small-voxel PET

		Optimal cut-off	Sensitivity	Specificity	Accuracy
SUV_max_	Standard-voxels	1.8	81%	95%	85%
	Small-voxels	2.6	78%	98%	84%
TB_ratio_	Standard-voxels	2.4	80%	82%	80%
	Small-voxels	3.3	84%	77%	82%

The use of small-voxel images requires higher SUV cut-offs for accurate lymph node characterization. Furthermore, SUV_max_ showed a higher performance as compared to TB_ratio_, with *p* = 0.04 for standard-voxels and *p* < 0.001 for small-voxels. However, the characterization performances were similar for standard- and small-voxel images, with *p* = 0.11 for SUV_max_ and *p* = 0.29 for TB_ratio_

*SUV*_*max*_ maximum standardized uptake value, *TB*_*ratio*_ ratio between the lymph node SUV_max_ and the lymph node background uptake, *PET* positron emission tomography

### Clinical examples

**Fig. 3:**
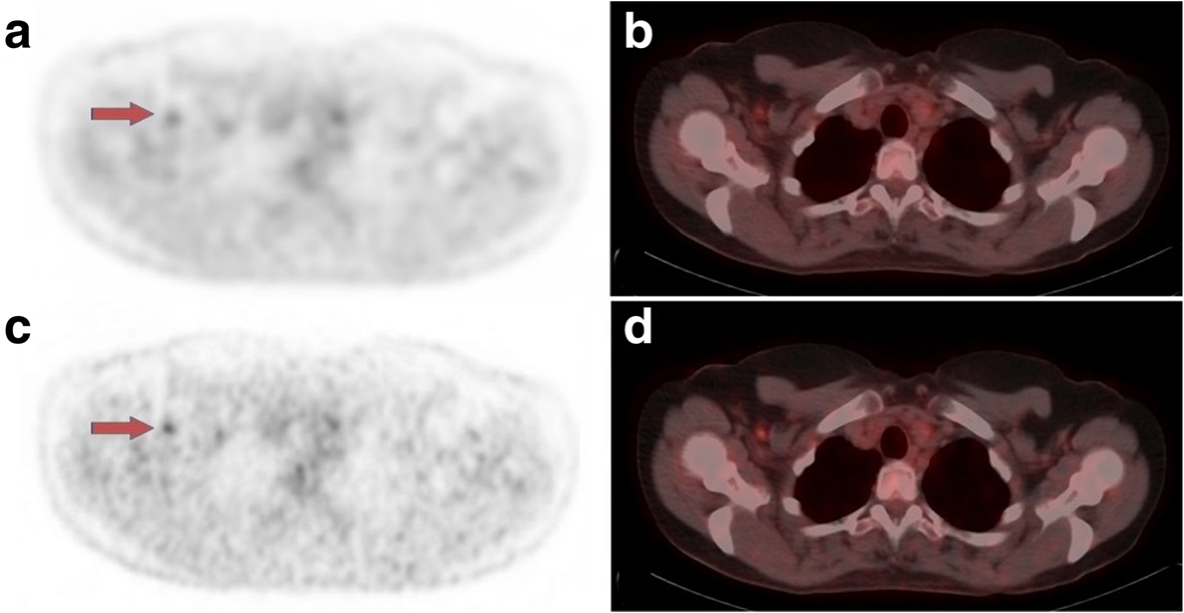
FDG-PET/CT images of a patient with proven breast cancer. **a** Axial PET image, standard-voxels. **b** Axial-fused PET/CT image, standard-voxels. **c** Axial PET image, small-voxels. **d** Axial-fused PET/CT image, small-voxels. SUV_max_ for this small lymph node (red arrows) increased from 2.1 on standard-voxel PET with visual score benign to SUV_max_ 3.3 on small-voxel PET and visual score malignant. On follow-up imaging after chemotherapy, this lymph node showed regression, which indicated that the lymph node was malignant. This confirmed the small-voxel score and the classification using the optimal SUV_max_ cut-off

Figure 3 shows FDG-PET/CT images from a patient with breast cancer. The visual score of the axillary lymph node altered from benign on standard-voxel PET/CT to malignant on small-voxel PET/CT. Furthermore, SUV_max_ of this lymph node increased by 57% on small-voxel images. Follow-up imaging showed that this lymph node responded to chemotherapy, which indicated that this lesion was malignant. This confirmed the small-voxel score and the classification by the optimal SUV_max_ cut-off shown in Table 4.

**Fig. 4:**
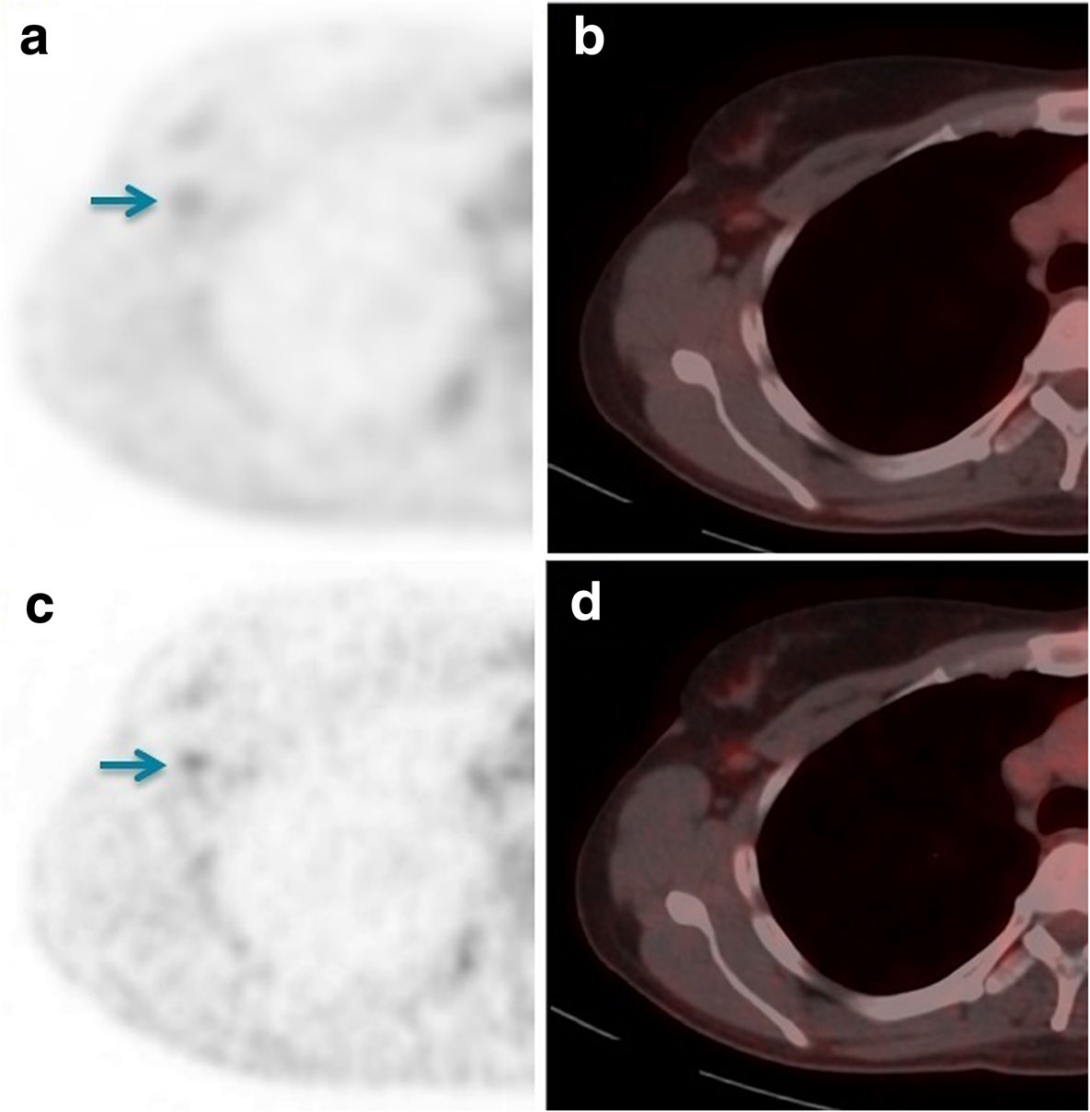
FDG-PET/CT images of a patient with proven breast cancer. **a** Axial PET image, standard-voxels. **b** Axial-fused PET/CT image, standard-voxels. **c** Axial PET image, small-voxels. **d** Axial-fused PET/CT image, small-voxels. For this lymph node, with short-axis diameter 5 mm in the right axillary region (blue arrows), SUV_max_ values were 1.4 and 2.3 (increase 64%) on standard- and small-voxel images, respectively. Furthermore, the visual scores were benign on standard-voxel PET and malignant on small-voxel PET. The sentinel node biopsy procedure did not reveal any malignancy. This indicates that this lymph node was benign, confirming the standard-voxel score and confirming the classification using the optimal SUV_max_ cut-off

Figure 4 shows FDG-PET/CT images from a breast cancer patient, with a small axillary lymph node. The visual score was benign on standard-voxel images while it was scored malignant on small-voxel images. In this case, SUV_max_ increased with 64% on small-voxel images. However, during sentinel node biopsy, no malignancy was found. This indicated that this lymph node was benign and the standard-voxel score was correct.

## Discussion

This study shows that small-voxel PET reconstructions improve the diagnostic sensitivity in the detection of lymph node metastases in breast cancer, at the expense of an impaired specificity. Based on visual evaluation of PET/CT images, the lymph node characterization accuracy did not change when using small-voxels instead of standard-voxels (*p* = 0.13). Nevertheless, limiting the analysis to malignant lymph nodes only, the small-voxel images were correct in 89% while the standard-voxel images provided concordant scores in only 76% of the cases (*p* < 0.001). This shows that the visual evaluation and detection of malignant axillary lymph nodes improves using small-voxel PET/CT. Contrarily, benign lymph nodes were more often correctly scored on standard-voxel images as compared to those on the small-voxel images (74 vs. 89%, *p* = 0.04).

Quantitatively, we found significant differences between benign and malignant lymph nodes for both parameters SUV_max_ and TB_ratio_ and both PET voxel sizes (2 × 2 × 2 mm^3^ and 4 × 4 × 4 mm^3^). Furthermore, the use of small-voxels resulted in higher FDG-uptake values of typically 40% for both benign and malignant lymph nodes. This increase is comparable with previously published values [10].

This study shows that the visual evaluation of malignant lymph nodes results in a higher accuracy and sensitivity when using small-voxel PET/CT. However, when looking quantitatively, both reconstruction voxel types can be used interchangeably for accurate loco-regional lymph node characterization. Furthermore, across all lymph nodes, both the quantitative and visual evaluation showed comparable performances in disease characterization. Moreover, when using small-voxel reconstructions, PET readers have to be aware of the risk of false-positives and the need of higher cut-off values to distinguish benign from malignant nodes. In the past, all visually FDG-positive axillary lymph nodes were considered as metastatic, because that approach resulted in high specificity [11, 12]. However, we demonstrated that on small-voxel PET images, benign loco-regional lymph nodes can also show some increased FDG-uptake. In the literature, this phenomenon has been described as well for the visualisation of benign nodes on PET images that incorporated point-spread function (PSF) modelling [19, 20]. Clinically, the main feature of small-voxel PET is the ability to detect more malignant axillary lymph nodes. This could lead to a higher disease stage and thereby potentially changes treatment. For example when the N-stage based on PET changes from N1 to N2, this could give rise to treat the patient with neo-adjuvant chemotherapy before surgery. Generally, with an improved detection of malignant axillary lymph nodes, the role of PET as an additional tool in the treatment decision plan can be further extended.

Between the two parameters, SUV_max_ and TB_ratio_, which were evaluated in this study, we found a significant difference in clinical performance. For both voxel reconstruction types, the SUV_max_ parameter was significantly better in distinguishing benign from malignant lymph nodes. This indicates that during quantitative PET evaluation, it is not required to take the surrounding background uptake around the lymph node into account for accurate characterization of loco-regional lymph nodes.

Despite the use of a small-voxel PET reconstruction, the detection rate with FDG-PET/CT for small metastases remains restricted. In our study, 21 out of 230 lymph nodes were not visualised on PET/CT images but were only found during the histological procedures after surgery. It is very likely that the size and/or the metabolic volume of those undetected lymph node metastasis was not large enough to be visualised with PET [2]. Currently, several developments in PET technology are taking place to further improve the diagnostic performance of PET/CT systems [21]. Together with the development of more specific radionuclide tracers for breast cancer, this may further improve the detection rate of PET/CT [22].

A limitation of this study is that the analysis was based on PET images together with low-dose CT only. Possibly, we did not incorporate all additional findings in case a diagnostic contrast-enhanced CT was obtained. It is likely that adding more CT aspects of lymph nodes, such as spherical shape and the absence of intra-nodal fat, will further improve the sensitivity, specifically of small-voxel reconstructions, and further reduce the false-positive rate. Moreover, there were some differences in visual scores between the two observers as consensus scoring was needed in 20% of the cases. This can be partly explained by the use of a five-point scoring system, which easily introduces small differences in visual scores between observers. Furthermore, the lack of a gold standard for the relatively new small-voxel PET images may have led to differences between observers. There is a learning curve involved in this new detailed method of reading the axilla in breast cancer patients. Also, possibly, results will improve by rigorous comparison with non-involved small nodes in the contralateral axilla as a normal reference.

The present study has some other limitations. The retrospective study design may have led to some bias in our study population, since only patients with a known lymph node status were included in this study. Additionally, since not all patients had a lymph node dissection, some microscopic nodes may have been missed in some patients. Moreover, the final diagnosis per lymph node in this study was based on three different references: histological data, FU imaging and additional imaging. Although the value of additional imaging as a reference method can be difficult, the number of final diagnoses that was based on this method was very limited (1%). Moreover, FDG-PET may give a false-positive result, e.g. in case of an inflammation after biopsy which would also disappear after, but not due to, neo-adjuvant chemotherapy.

Some recommendations can be made for further research. The small-voxel reconstruction protocol could be further optimized to improve image quality and lesion detectability. Recently, Bellevre et al. [19] demonstrated that the use of PSF modelling improved the performance of axillary staging in breast cancer patients. A combination of small-voxels and PSF modelling could be explored. Furthermore, the number of iterations and subsets could be optimized [20, 23, 24]. Meanwhile, other evaluation methods could be considered. For example, the value of combined standard- and small-voxel PET in visual evaluation can be explored, to further improve the accuracy. Also, the use of SUV_peak_ could potentially decrease differences between both reconstructions as it is less influenced by image noise as compared to SUV_max_ [25]. Furthermore, the added value of the optimal SUV cut-offs as proposed in this study, onto the visual evaluation of small-voxel PET, can be studied. Also, optimal SUV cut-offs can be different in other types of breast cancers and may be influenced when incorporating data on the tumour proliferation index (ki67) or the hormone receptor status.

Apart from this, it can be interesting to study the clinical impact of small-voxel images in other body regions and for other diseases. For example, we previously evaluated the impact of a state-of-the-art PET/CT scanner on the visualisation and quantification of adrenal glands, using a standard-voxel reconstruction [26]. This could be extended towards an evaluation of the impact of a small-voxel reconstruction on adrenal gland quantification and classification with FDG-PET/CT.

## Conclusions

We evaluated the diagnostic properties of a small-voxel reconstruction for loco-regional lymph node staging in 69 patients with breast cancer, using state-of-the-art TOF FDG-PET/CT. The use of small-voxels improves the sensitivity of visual PET/CT evaluation in malignant lymph nodes, as compared to standard-voxel analysis. However, it also introduced more false-positive results for benign nodes. Across all nodes, differences in accuracy were non-significant. Quantitatively, a small-voxel reconstruction implicates higher SUV cut-off values when differentiating benign from malignant axillary lymph nodes. In case small-voxel images are used, readers have to adapt their reference standards visually and quantitatively.

## Abbreviations

AUC: Area under the curve
CI: Confidence interval
CT: Computed tomography
EANM: European Association of Nuclear Medicine
EARL: EANM research Ltd.
FDG: Fluor-18 fluordeoxyglucose
FU: Follow-up
LYSO: Lutetium yttrium orthosilicate
MRI: Magnetic resonance imaging
NM: Nuclear medicine
PET: Positron emission tomography
ROC: Receiver operator curve
ROI: Region of interest
SD: Standard deviation
Small-voxels: 2 × 2 × 2 mm^3^ voxels
SNR: Signal-to-noise ratio
Standard-voxels: 4 × 4 × 4 mm^3^ voxels
SUV: Standardized uptake value
SUV_background_: SUV in the background
SUV_max_: Maximum SUV
TB_ratio_: Ratio between SUV_max_ and SUV_background_
TOF: Time of flight
